# Associative memory model with long-tail-distributed Hebbian synaptic connections

**DOI:** 10.3389/fncom.2012.00102

**Published:** 2013-02-07

**Authors:** Naoki Hiratani, Jun-Nosuke Teramae, Tomoki Fukai

**Affiliations:** ^1^Department of Complexity Science and Engineering, Graduate School of Frontier Sciences, The University of TokyoKashiwa, Japan; ^2^Laboratory for Neural Circuit Theory, RIKEN Brain Science InstituteWako, Japan; ^3^PRESTO, JSTKawaguchi, Saitama, Japan; ^4^CREST, JSTKawaguchi, Saitama, Japan

**Keywords:** integrate-and-fire, storage capacity, stochastic resonance, hippocampus, attractor, mean-field

## Abstract

The postsynaptic potentials of pyramidal neurons have a non-Gaussian amplitude distribution with a heavy tail in both hippocampus and neocortex. Such distributions of synaptic weights were recently shown to generate spontaneous internal noise optimal for spike propagation in recurrent cortical circuits. However, whether this internal noise generation by heavy-tailed weight distributions is possible for and beneficial to other computational functions remains unknown. To clarify this point, we construct an associative memory (AM) network model of spiking neurons that stores multiple memory patterns in a connection matrix with a lognormal weight distribution. In AM networks, non-retrieved memory patterns generate a cross-talk noise that severely disturbs memory recall. We demonstrate that neurons encoding a retrieved memory pattern and those encoding non-retrieved memory patterns have different subthreshold membrane-potential distributions in our model. Consequently, the probability of responding to inputs at strong synapses increases for the encoding neurons, whereas it decreases for the non-encoding neurons. Our results imply that heavy-tailed distributions of connection weights can generate noise useful for AM recall.

## Introduction

The organization of neuronal wiring determines the flow of information in neural circuits and hence has significant implications for functions of the circuits. A number of recent studies revealed the non-random features of neuronal wiring in cortical circuits (Markram, 1997; Kalisman et al., 2005; Song et al., 2005; Yoshimura et al., 2005; Koulakov et al., 2009; Lefort et al., 2009; Yassin et al., 2010; Perin et al., 2011). These features include not only the complex topology of neuronal wiring, but also the non-Gaussian nature of the distributions of amplitudes of excitatory postsynaptic potentials (EPSPs), which is typically represented by the presence of long tails (also called “heavy tails”) in the distributions. In fact, the amplitude distributions of EPSPs are well described by lognormal distributions in both neocortex (Song et al., 2005; Lefort et al., 2009) and hippocampus (Ikegaya et al., 2013). In reality, the amplitude of EPSP between cortical neurons may represent the total strength of multiple synaptic contacts made by one of the neurons on the other. However, hereafter we simply call the EPSP amplitude the “synaptic weight” from one neuron to the other.

A lognormal weight distribution implies that a small number of very strong connections are present in local cortical circuits and carry a large amount of the total weight on a cortical neuron, while the majority of synapses are weak (Holmgren et al., 2003; Binzegger et al., 2004). Such EPSP-amplitude distributions of AMPA receptor-mediated synapses significantly influence the dynamical properties of stable network states (Koulakov et al., 2009; Roxin et al., 2011). In particular, we have recently shown that spontaneous cortical activity emerges from the cooperation between strong-sparse and weak-dense (SSWD) synapses in a heavy-tailed EPSP-amplitude distribution of AMPA recurrent synapses (Teramae et al., 2012). Such a recurrent network can generate internal noise optimal for stochastic resonance effects on spike-based communications between neurons. Moreover, lognormally-connected recurrent networks combined with highly non-random properties of cortical synaptic connections (Prill et al., 2005; Song et al., 2005; Perin et al., 2011) generate working memory-like bistable network states (Klinshov et al., unpublished data). These results seem to indicate that heavy-tailed weight distributions play active roles in stochastic cortical information processing including the pattern-recall operation in the hippocampus. However, these roles remain largely unknown.

Hippocampal CA3 has sparse recurrent excitatory connections and is thought to perform a pattern completion operation in memory recall (Nakazawa et al., 2002). Properties of pattern completion, such as storage capacity and memory retrieval dynamics, have been extensively studied in the statistical mechanics of Hopfield associative memory (AM) network and its variants of formal neurons with binary or analog outputs (Hopfield, 1982, 1984; Amit et al., 1985; Derrida et al., 1987; Tsodyks and Feigel'man, 1988; Shiino and Fukai, 1992; Coolen and Sherrington, 1993; Okada, 1995). AM models of spiking neurons have also been studied although they generally exhibit a much poorer ability for memory storage than models of binary or analog neurons (Gerstner and Hemmen, 1992; Lansner and Fransén, 1992; Treves, 1993; Amit and Brunel, 1997; Maass and Natschläger, 1997; Sommer and Wennekers, 2001; Curti et al., 2004; Latham and Nirenberg, 2004; Aviel et al., 2005; Roudi and Latham, 2007). In most of the previous models, the weights of synaptic connections typically obey a Gaussian distribution, particularly when the number of embedded patterns is extensively large. However, since the amplitudes of EPSPs between pyramidal cells were shown to obey a heavy-tailed distribution in CA3 (Ikegaya et al., 2013), here we construct AM network models of spiking neurons having lognormal weight distributions of recurrent connections and investigate possible implications of such non-Gaussian distributions in the memory retrieval dynamics. In particular, we explore the possibility that such a network can generate internal noise useful for the retrieval of an embedded memory pattern.

## Materials and methods

We developed a recurrent network model of integrate-and-fire neurons that stores information on a multiple number of memory patterns in the weights of excitatory synaptic connections obeying a lognormal distribution. Extremely strong synapses are rare in such a weight distribution and the majority of synapses are weak. Therefore, we may term neural networks with long-tailed synaptic weight distributions “SSWD networks.”

### Network dynamics

**Figure 1:**
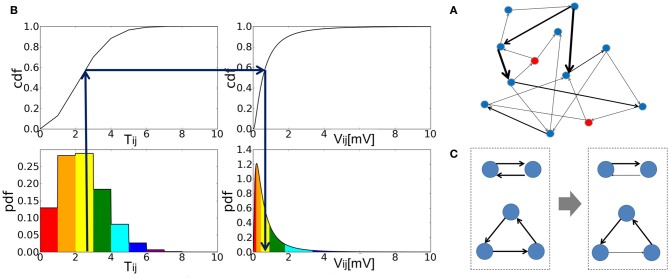
**The construction of an associative memory network with strong-sparse and weak-dense synapses. (A)** Schematic illustration of strong-sparse weak-dense connected network. **(B)** The cumulative distribution function (cdf) was calculated from the probability distribution of the elements of the Hebbian connection matrix *T*_*ij*_ (*left*). Then, the EPSP amplitude *V*_*ij*_ of this synaptic connection was determined by a one-to-one mapping between the cdf of *T*_*ij*_ and that of the target lognormal distribution for *V*_*ij*_ (*right*). **(C)** Reciprocal and triangle connections are schematically shown before (*left*) and after rewiring (*right*). In both cases, one of the strong connections are chosen randomly and eliminated.

The model consists of *N*_*E*_ excitatory neurons and *N*_*I*_ inhibitory neurons. The excitatory neurons are connected with each other by a lognormally weight-distributed Hebbian-type synaptic matrix that is generated from random memory patterns, and each inhibitory neuron is connected randomly with the excitatory neurons and the other inhibitory neurons (Figure 1A). The neural dynamics in this recurrent network is described by conductance-based leaky integrate-and-fire neurons as:

(1) $$\frac{dv_{i}}{dt}=-\frac{1}{\tau_{m}}\left(v_{i}-V_{L}\right)-g_{i}^{E}\left(v_{i}-V_{E}\right)-g_{i}^{I}\left(v_{i}-V_{I}\right)$$

where *v*_*i*_ is the membrane potential of neuron *i*, τ_*m*_ is the membrane time constant, and *V*_*E*_, *V*_*I*_, and *V*_*L*_ are the reversal potentials of AMPA-receptor-mediated excitatory synaptic current, inhibitory synaptic current, and leak current, respectively. The conductances of excitatory and inhibitory synapses are measured in units of [time^−1^] and obey.

(2) $$\frac{dg_{i}^{X}}{dt}=-\frac{g_{i}^{X}}{\tau_{s}}+\sum_{j}^{N_{X}}c_{ij}^{XY}G_{ij}^{XY}\sum_{s_{j}}\delta \left(t-s_{j}^{X}-d_{ij}^{XY}\right)\text{​},\;X,Y=E,I$$

where τ^*E,I*^_*s*_ is the decay constant of excitatory or inhibitory synaptic current, *s*^*E,I*^_*j*_ is the spike times of excitatory or inhibitory neuron *j, d*^*XY*^_*ij*_ is delay from neuron *j* to *i*, and the element of the adjacent matrix *c*^*XY*^_*ij*_ is 1 if neurons *j* and *i* are connected and otherwise 0. Indices *X* and *Y* indicate whether pre- or post-neurons are excitatory or inhibitory. The maximum conductance *G*^*EE*^ of excitatory-to-excitatory synapses was determined such that the amplitudes of their EPSPs obey a lognormal distribution *N*(μ, σ^2^), with parameters μ and σ^2^ being the mean and variance of the normal distribution. Other types of conductance (*E*-to-*I, I*-to-*E, I*-to-*I*) were fixed at constant values.

Excitatory synapses fail to transmit presynaptic spikes to postsynaptic neurons with a certain probability. We defined the failure probability as *V*_*a*_/(*V*_*a*_ + *V*_*in*_) in terms of the threshold potential *V*_*a*_ for a synapse with an EPSP amplitude of *V*_*in*_. Each neuron outputs a spike when its membrane potential reaches threshold *V*_*th*_, then the membrane potential is reset to *V*_*L*_ after the refractory period τ_*ref*_.

We used the following values of parameters in the present numerical simulations. The number of excitatory neurons *N*_*E*_ = 10,000 and that of inhibitory neurons *N*_*I*_ = 2000. The connection probabilities, i.e., the probabilities of finding a non-vanishing element in the adjacent matrices, are *c*_*E*_ = 0.1 and *c*_*I*_ = 0.5 for excitatory and inhibitory synapses, respectively. The decay time constants are τ^*E*^_*m*_ = 20.0 [ms] and τ^*I*^_*m*_ = 10.0 [ms] for excitatory and inhibitory membrane decay, and τ_*s*_ = 2.0 [ms] for synaptic decay. We chose average delay of synaptic inputs as τ^*E*^_*d*_ = 2.0 [ms] for excitatory synaptic connections, and τ^*I*^_*d*_ = 1.0 [ms] for inhibitory connections. The refractory period is τ_*ref*_ = 1.0 [ms]. For membrane potential parameters, spike threshold is *V*_*th*_ = −50.0 [mV], reversal potential of leak current is *V*_*L*_ = −70.0 [mV], and reversal potential of postsynaptic currents are *V*_*E*_ = −0.0 [mV] and *V*_*I*_ = −80.0 [mV] for excitatory and inhibitory, respectively. Threshold for synaptic transmission failure is *V*_*a*_ = 0.1 [mV]. The weights of synaptic inputs are *G*^*EI*^ = 0.017, *G*^*IE*^ = 0.0018, *G*^*II*^ = 0.0025 for E-to-I, I-to-E, I-to-I connections. For E-to-E connections, parameters of lognormal distribution are σ = 1.0, and μ = σ^2^ + log 0.2, and upper limit of EPSP is *V*_*max*_ = 20.0 [mV]. Computer codes are written in C++ and dynamical equations are solved by using Euler methods with a time step of *h* = 0.01 [ms].

### Synaptic connections

We embedded mutually-independent random memory patterns into *E*-to-*E* connections as follows. First, we created random binary patterns of 0 and 1 {ξ^μ^_*i*_}^1,2,…,*p*^_*i* = 1,2,…, *N*_*E*__ according to:

(3) $$\text{Prob​}\left[\xi_{i}^{\mu }=1\right]=a,\text{ Prob}\left[\xi_{i}^{\mu }=0\right]=1-a$$

where *p* is the total number of embedded patterns and *a* is the sparseness of these patterns. In this paper, we introduced a constraint as ∑_*i*_ξ^μ^_*i*_ = *aN*_*E*_ to suppress the non-homogeneity across different memory patterns.

The conventional weight matrix of AM network model is determined by the local Hebbian rule as ∑_μ_ ξ^μ^_*i*_ ξ^μ^_*j*_. However, here we want to create EPSP amplitudes that obey a lognormal distribution, while the conventional weight matrix obeys a binomial distribution. In order to accomplish this, we introduced a continuous weight matrix *T*_*ij*_ as shown below:

(4) $$T_{ij}=\sum_{\mu \;=\;1}^{p}\xi_{i}^{\mu }\xi_{j}^{\mu }+\zeta_{ij}$$

where ζ_*ij*_ ∈ [0,1) is a random variable to make the distribution of *T*_*ij*_ continuous. The second term in Equation 4 is crucial for the genesis of spontaneous activity when the network stores only a small number of patterns and hence the first term vanishes between most neuron pairs. Then, we determined EPSP *V*_*ij*_ between excitatory neurons *i* and *j* so that the cumulative frequency of *V*_*ij*_ may coincide with that of *T*_*ij*_ (Figure 1B). Defining the set of memory patterns supported by neuron *i* as μ_*i*_ = {μ|ξ^μ^_*i*_ = 1}, we can express *T*_*ij*_ as *T*_*ij*_ = ∑_μ ∈ μ_*i*__ ξ^μ^_*i*_ + ζ_*ij*_ where μ_*i*_ contains *p*_*i*_ = ∑_μ_ ξ^μ^_*i*_ memory patterns. Thus, the cumulative function of *T*_*ij*_ is written as:

$$\text{Prob}\left[T_{ij}\leq x\right]=\sum_{q\;=\;0}^{\tilde{x}-1}h\left(p_{i},q\right)+\left(x-\tilde{x}\right)h\left(p_{i},\tilde{x}\right)$$

in terms of *h*(*p*_*i*_,*q*) =_*p*_*i*__ *C*_*q*_*a*^*q*^ (1 − *a*)^*p*_*i*_ − *q*^, where $\tilde{x}$ is the maximum integer below *x*. On the other hand, the cumulative distribution of *V*_*ij*_ is written as:

$$\begin{array}{l} \text{Prob}\left[V_{ij}\leq y\right]=\frac{1}{2Z_{v}}\left(1+\text{erf}\left[\frac{1}{\sqrt{2\sigma }}\left(\log y-\mu \right)\right]\right)\text{​},\\ \;Z_{v}=\frac{1}{2}\left(1+\text{erf}\left[\frac{1}{2\sigma }(\log V_{max}-\mu )\right]\right)\text{​}. \end{array}$$

where *V*_*max*_ is the upper bound for *V*_*ij*_. Therefore, the map *x* → *y* is given as follows:

(5) $$y=\exp\left[\mu +\sqrt{2}\sigma {\text{erf}}^{-1}\left(2Z_{v}\text{Prob}\left[x\leq T_{ij}\right]-1\right)\right]\text{​},$$

for the pair of neurons *i* and *j*.

If some excitatory neurons send too strong excitatory outputs to other neurons, these neurons can be a potential hazard to the stability of network dynamics. To prevent the appearance of such hubs, we normalized each synaptic weight over presynaptic neurons as:

(6) $$V_{ij}=y/Z_{j},\;Z_{j}=\exp\left(\left(p_{j}-pa\right)/pa\right)\text{​},$$

where the normalization factor *Z*_*j*_ is greater or smaller than unity if the number of memory patterns encoded by neuron *j* is larger or smaller than the expectation value, respectively.

As in many other AM network models, the connection matrix constructed above is symmetric. In networks of binary or monotonic analog neurons, symmetric synaptic connections induce a “down-hill” motion in the time evolution of network states, making the retrieval of memory patterns possible (Hopfield, 1982, 1984). The connection matrix shown in Equation 6 generates a small number of strong (typically, EPSP >3 mV) reciprocal connections and triangle motifs. Though the over-representations of such motifs have been known in cortical circuits (Song et al., 2005; Perin et al., 2011), here such motifs form strong excitatory loops due to the symmetry of the connection matrix. Therefore, a small number of neurons belonging to the motifs are activated very strongly and tend to burst at very high frequencies. To avoid this unrealistic situation, we weakened one of the edges in all strong loops (Figure 1C). To be precise, we searched all “strong” reciprocal excitatory connections that generate EPSPs larger than a threshold value *V*_*u*_ in both directions. Then, we selected one of them randomly and assigned to it a new EPSP that is smaller than *V*_*l*_. In addition, we rewired “strong” triangle connections in a similar way such that they may not form a circular triangle because neurons in the closed loop structure tend to give high-frequency repetitive bursts in numerical simulations. In this rewiring, the lower limit of strong EPSP *V*_*l*_ is 3.0 [mV] and the upper limit of weak EPSP *V*_*u*_ is 1.0 [mV].

### Numerical simulations

We conducted numerical simulations of the model for various values of parameters. To initiate spontaneous activity, we applied external Poisson spike trains at 10 Hz to all neurons for the initial 100 ms of each simulation trial and evoked EPSPs with the amplitude of about 10 mV. Once spontaneous activity is triggered, the network continues to show autonomous firing without any external input. This spontaneous firing state may be regarded as the resting state of the network. Depending on the values of parameters, the stability of spontaneous activity is not robust enough and the network may exhibit a further spontaneous transition from the resting state due to the intrinsic noise generated by reverberating synaptic input. As explained in the Results, the lifetime of spontaneous activity depends significantly on the memory load. If the network remained in the resting state for more than 500 ms after the initiation of spontaneous activity, we stimulated neurons encoding a memory pattern with external input of the duration 10 ms and the frequency 100 Hz. We may regard this input as a cue signal for memory retrieval.

### Pattern overlaps

We define a macroscopic order parameter to measure overlaps between the network state and memory patterns as:

(7) $$K_{\mu }=\frac{1}{aN_{E}r_{\mu }}\sum_{i}\left(\xi_{i}^{\mu }-a\right)\left(r_{i}-r_{E}\right)$$

where *r*_*E*_ = (1/*N*_*E*_)∑_*i*_ *r*_*i*_ and *r*_μ_ = (1/*aN*_*E*_) ∑_*i*_ ξ^μ^_*i*_*r*_*i*_, and *r*_*i*_ is the firing rate of the *i*-th excitatory neuron. In numerical simulations, the firing rate was calculated from spike data in the interval of 800 < *t* < 1100 [ms]. Spike data during 610 < *t* < 800 [ms] was not used because the system is in a transient state. The pattern overlap with the retrieved memory pattern may be called “the retrieval rate.”

The retrieval rate is written as *K* = 1 − *r*_*E*_/*r*_μ_ (this expression is exact due to the restriction on the total number of non-vanishing components in each pattern), therefore, *K* ≅ 1 when the system is in the retrieval state and *K* ≅ 0 in the resting state. The retrieval rate *K* = 0 when (a) all neurons are completely silent, (b) when a non-target memory pattern is retrieved or (c) when the average firing rate of the retrieval state becomes extremely high. The case (a) sometimes occurs when memory patterns are not sparse enough (typically, *a* ≥ 0.15) and the number of stored patterns is large. The case (b) occurs regardless of the sparseness parameter *a* when a small number of patterns are stored. Note that the target pattern is not stable in this case. In case (c), we regarded memory retrieval as unsuccessful since PR neurons fired at unrealistically high firing rates (typically, >70Hz). Therefore, we set *K* = 0 without calculating the right-hand side of Equation (7) although there could be a different treatment for this case. Case (c) typically occurs when memory patterns are sparse (*a* ≤ 0.10) and the number of stored patterns is relatively small, that is, in the lower left off-diagonal region of the parameter space shown in Figures 1B and 7B.

### Mean-field approximation

**Figure 2:**
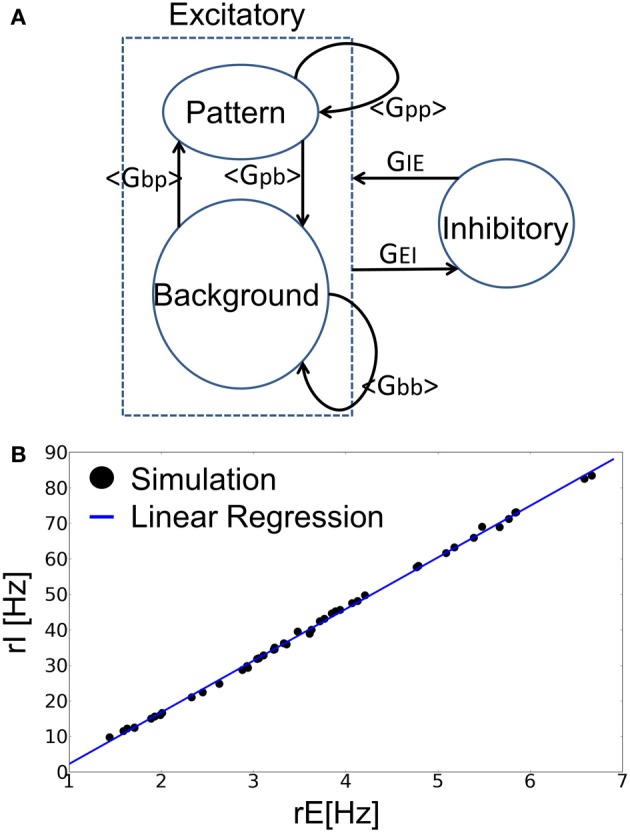
**Mean-field analysis of the recurrent neural network. (A)** The approximate treatment employed in the present analysis is schematically illustrated. **(B)** The relation between *r*_*E*_ and *r*_*I*_ in the balanced state. The blue line is the linear regression of simulation results. Each data point (filled circle) shows the average firing rates of excitatory and inhibitory neurons in both spontaneous state and retrieval state at various values of the parameters *a* and *p*.

We analyze the retrieval of a representative memory pattern and regard the other patterns as a Gaussian white noise reservoir. Then, in this approximation the model consists of three neuronal populations, i.e., excitatory pattern-retrieval (PR) neurons, excitatory background (BG) neurons and inhibitory neurons (Figure 2A). In the equations below, indices *p* and *b* stand for PR and BG, respectively. Below, we explain the outline of the mean-field approximation and show the details of the analysis in Appendix. This approximation is valid when the total number of inputs to each neuron is sufficiently large and the total input is approximated to be the sum of a drift term and a fluctuation term. In addition, in order for a statistical approach to be valid, each memory pattern should contain a sufficiently large number of member neurons.

We first calculate the average synaptic input from PR neurons to PR neurons <*G*_*pp*_> as:

(8) $$\langle G_{pp}\rangle =\frac{1}{c_{E}\sum_{i}\sum_{j}\xi_{i}^{1}\xi_{j}^{1}}\sum_{i}\sum_{j}\xi_{i}^{1}\xi_{j}^{1}F_{p}\left[\frac{V_{ij}}{V_{a}+V_{ij}}\right]G_{ij}^{EE},$$

where *F*_*p*_ is a binary probability variable that is defined as Prob [*F*_*p*_(*X*) = 1] = *X*, Prob [*F*_*p*_ (*X*) = 0] = 1 − *X*. The average synaptic inputs <*G*_*bp*_>, <*G*_*pb*_> and <*G*_*bb*_> from BG to PR, PR to BG, and BG to BG, respectively, are also obtained in similar manners. The average squared synaptic weights 〈*G*^2^_*qq*_〉 are calculated in the same manner. Since <*G*_*pp*_> is larger than the average synaptic input averaged over all neurons <*G*_*EE*_>, strong recurrent connections among PR neurons contribute significantly to sustaining network activity for the recalled memory pattern. <*G*_*bp*_> is typically smaller than <*G*_*EE*_> since each excitatory neuron receives almost the same average input. Other connections <*G*_*pb*_> and <*G*_*bb*_> are roughly the same as <*G*_*EE*_> in the region of parameter values under consideration.

If the synaptic weight matrix was a linear superposition of memory patterns, *G*_*ij*_ ∝ ∑_μ_ ξ^μ^_*i*_ξ^μ^_*j*_, we could separate the contribution of retrieved pattern and that of non-retrieved patterns to the mean field equation and analytically evaluate the storage capacity. In our model, however, this separation is quite difficult due to a non-linear transformation from *T*_*ij*_ = ∑_μ_ξ^μ^_*i*_ξ^μ^_*j*_ to the lognormal synaptic weight matrix *G*_*ij*_. Nonetheless, we can qualitatively understand how the average weight depends on the number of stored memory patterns. Noting that the synaptic conductance *G*^*EE*^_*ij*_ is approximately proportional to the EPSP amplitude which obeys a lognormal distribution, we can approximately express the synaptic weight matrix as *G*^*EE*^_*ij*_ ≈ (γ/*Z*) exp(β ∑_μ_ξ^μ^_*i*_ξ^μ^_*j*_) for infinitely large *p*, where $\beta =\sigma /\sqrt{pa^{2}\left(1-a^{2}\right)}$ and *Z* = exp(β*pa*^2^ − μ). Thus, we obtain:

$$\begin{array}{l} \langle G_{pp}\rangle \approx \frac{1}{a^{2}}\langle \xi_{i}^{1}\xi_{j}^{1}G_{ij}^{EE}\rangle =\frac{1}{a^{2}}\langle \xi_{i}^{1}\xi_{j}^{1}\frac{\gamma }{Z}\prod_{\mu }\left[1+\left(e^{\beta }-1\right)\xi_{i}^{\mu }\xi_{j}^{\mu }\right]\rangle \\ \;=\frac{1}{a^{2}}\langle e^{\beta }\xi_{i}^{1}\xi_{j}^{1}\frac{\gamma }{Z}\prod_{\mu \;\neq \;1}\left[1+\left(e^{\beta }-1\right)\xi_{i}^{\mu }\xi_{j}^{\mu }\right]\rangle \approx e^{\beta }\langle G_{ij}^{EE}\rangle , \end{array}$$

or $<G_{pp}>/<G_{ij}^{EE}>\propto \exp(1/\sqrt{p})$, which implies that the relative magnitude of reverberating synaptic input among PR neurons decreases with an increase in the number of stored patterns. Although the above approximation is not accurate for finite values of *p*, the asymptotic behavior of <*G*_*pp*_> explains how the storage capacity is determined in the present model.

Then, denoting the average firing rate of each neural population as *r*_*p*_, *r*_*b*_, and *r*_*i*_, we obtain the following approximate relationship in the balanced state:

(9) $$r_{I}=r_{Io}+k_{o}r_{E},$$

where *r*_*E*_ = *ar*_*p*_ + (1–*a*)*r*_*b*_ is the average firing rate of all excitatory neurons. Numerical simulations confirmed the validity of this relationship (Figure 2B). Then, the time evolution of excitatory neurons is described by LIF neurons as follows:

$$\begin{array}{l} \frac{dv_{i}^{q}}{dt}=-\frac{1}{\tau_{m}^{E}}\left(v_{i}^{q}-V_{L}\right)-g_{i}^{Eq}\left(v_{i}^{q}-V_{E}\right)-g_{i}^{IE}\left(v_{i}^{q}-V_{I}\right)\\ \frac{dg_{i}^{Eq}}{dt}=-\frac{g_{i}^{Eq}}{\tau_{S}}+\sum_{j}^{N_{E}}c_{ij}^{EE}G_{ij}^{Eq}\sum_{s_{j}}\delta (t-s_{j}-d_{ij})\\ \frac{dg_{i}^{IE}}{dt}=-\frac{g_{i}^{IE}}{\tau_{S}}+\sum_{j}^{N_{I}}c_{ij}^{IE}G_{ij}^{IE}\sum_{s_{j}}\delta (t-s_{j}-d_{ij}) \end{array}$$

where *q* = *p* or *b*.

As in the previous model (Teramae et al., 2012), we treated excitatory synaptic inputs to each neuron as the sum of Gaussian noise generated by weak-dense synaptic inputs and *m* sparse-strong synaptic inputs:

$$g_{i}^{Eq}=\langle g^{Eq}\rangle +\eta_{i}^{Eq},\;\frac{d\eta_{i}^{Eq}}{dt}=-\frac{\eta_{i}^{Eq}}{\tau_{s}}+s^{Eq}\zeta_{i}(t),$$

where ζ_*i*_ is a normalized Gaussian random variable with mean zero. Then the average and variance of the synaptic conductance *g*^*Eq*^_*i*_ can be written as:

(10) $$\begin{array}{l} \langle g^{Ep}\rangle =t_{s}c_{E}N_{E}\left(a\langle G_{pp}\rangle r_{p}+(1-a)\langle G_{bp}\rangle r_{b}\right),\\ \langle g^{Eb}\rangle =t_{s}c_{E}N_{E}\left(a\langle G_{pb}\rangle r_{p}+(1-a)\langle G_{bb}\rangle r_{b}\right)\\ \;≅t_{s}c_{E}N_{E}\langle G_{EE}\rangle r_{E}, \end{array}$$

(11) $$\begin{array}{l} {\left(s^{Ep}\right)}^{2}=c_{E}N_{E}\left(a\langle G_{pp}^{2}\rangle r_{p}+(1-a)\langle G_{bp}^{2}\rangle r_{b}\right),\\ {\left(s^{Eb}\right)}^{2}=c_{E}N_{E}\left(a\langle G_{pb}^{2}\rangle r_{p}+(1-a)\langle G_{bb}^{2}\rangle r_{b}\right)\\ \;≅c_{E}N_{E}\langle G_{EE}^{2}\rangle r_{E}. \end{array}$$

In Equations (10) and (11), we assumed that average inputs to BG neurons from PR and BG neurons are approximately the same for the two presynaptic neuron categories. Similarly, the fluctuating inhibitory synaptic input *g*^*IE*^_*i*_ is approximated as:

$$g_{i}^{IE}=\langle g^{IE}\rangle +\eta_{i}^{IE},\;\frac{d\eta_{i}^{IE}}{dt}≅-\frac{\eta_{i}^{IE}}{\tau_{s}}+s^{IE}\zeta_{i}\left(t\right),$$

where 〈*g*^*IE*^_*i*_〉 = *t*_*s*_*c*_*I*_*N*_*I*_*G*_*IE*_*r*_*I*_, (*s*^*EI*^)^2^ = *c*_*I*_*N*_*I*_*G*^2^_*IE*_*r*_*I*_ and η^*IE*^ is Gaussian white noise. Therefore, the membrane potential *v*^*q*^_*i*_ obeys

$$\begin{array}{l} \frac{dv_{i}^{q}}{dt}≅-\frac{v_{i}^{q}-V_{o}^{q}}{\tau_{e}^{q}}-\eta_{i}^{Eq}(V_{o}^{q}-V_{E})-\eta_{i}^{IE}(V_{o}^{q}-V_{I}),\\ \;\;\tau_{e}^{q}={\left[1/t_{m}^{E}+\langle g^{IE}\rangle +\langle g^{Eq}\rangle \right]}^{-1},\\ \;V_{o}^{q}=\tau_{e}^{q}[V_{L}/t_{m}^{E}+\langle g^{Eq}\rangle V_{E}+\langle g^{IE}\rangle V_{I}]. \end{array}$$

In the right-hand side of the first equation, we replaced the membrane potential with its average value. For this approximation to be valid, the variance of the membrane potential of excitatory neurons should be smaller than |*V*^*q*^_*o*_ − *V*_*E*_| and |*V*^*q*^_*o*_ − *V*_*I*_|. Otherwise, the fluctuation term may depend on the membrane potential and the analytic calculation of firnig rate will be difficult. The above equations represent Kramer's equation with a linear force. Taking the contributions from strong-sparse synapses ${\hat{r}}_{q}^{2}$ into account, we obtain equations for the time evolution of *r*_*p*_ and *r*_*b*_ as follows (Risken, 1996; Brunel, 2000; Teramae et al., 2012):

(12) $$\frac{dr_{q}}{dt}=-\frac{1}{\tau_{e}^{q}}\left(r_{q}-{\hat{r}}_{q}\left(r_{p,}r_{b}\right)\right),$$

(13) $${\hat{r}}_{q}={\hat{r}}_{q}^{1}+{\hat{r}}_{q}^{2},$$

(14) $${\hat{r}}_{q}^{1}=\frac{1}{2\pi \sqrt{\tau_{e}^{q}\tau_{s}}}\exp\left[-\frac{1}{2\sigma_{q}^{2}}{\left(V_{th}-V_{o}^{q}\right)}^{2}\right],$$

(15) $$\begin{array}{c} {\hat{r}}_{q}^{2}=\frac{N_{s}r_{E}}{2}(\text{erf}\left[\frac{1}{\sqrt{2}\sigma_{q}}\left(V_{th}-V_{o}^{q}\right)\right]\\ -\text{erf}\left[\frac{1}{\sqrt{2}\sigma_{q}}\left(V_{th}-V_{o}^{q}-V_{s}\right)\right]) \end{array}$$

where *q* = *p* or *b*, and the variances are given as:

$$\begin{array}{l} \sigma_{q}^{2}={\left[\frac{1}{\tau_{e}^{q}}+\frac{1}{\tau_{s}}\right]}^{-1}\frac{\tau_{e}^{q}\tau_{s}}{2}\left[\sigma_{Eq}^{2}+\sigma_{Iq}^{2}\right],\\ \sigma_{Eq}^{2}={\left(s^{Eq}\right)}^{2}{\left(V_{o}^{q}-V_{E}\right)}^{2},\\ \sigma_{Iq}^{2}={\left(s^{Iq}\right)}^{2}{\left(V_{o}^{q}-V_{I}\right)}^{2}. \end{array}$$

By solving Equations (12)–(15), we derived the various properties of the present model reported in the main text. The approximation by Kramer's equations is valid when the average membrane potential is sufficiently low and the probability distribution of the membrane potential over the threshold is negligibly small. The approximation is also not so accurate when the average firing rate of excitatory neurons is high since the membrane potential distribution is biased toward the threshold and spike trains are less irregular (in the cases of *p* = 120 and 125 in Figure 5E). We employed a linear approximation (Equation 9) for inhibitory neurons since their membrane potentials have a non-negligible probability density over the threshold and Kramer's equation is not accurate.

## Results

We conducted numerical simulations of the present model and explored its memory retrieval dynamics numerically and analytically. In numerical simulations, one of the stored patterns μ was excited by external input. We found that a heavy-tailed weight distribution of Hebbian synapses creates memory-specific subthreshold membrane potential fluctuations to regulate the stochastic dynamics of memory retrieval. In the rest of the paper, we call excitatory neurons encoding the retrieved pattern as PR (pattern retrieval) neurons, and those not encoding the pattern as BG neurons. For instance, when pattern 1 is retrieved (μ = 1), neuron *i* is a PR neuron if ξ^1^_*i*_ = 1 or a BG neuron if ξ^1^_*i*_ = 0.

**Figure 3:**
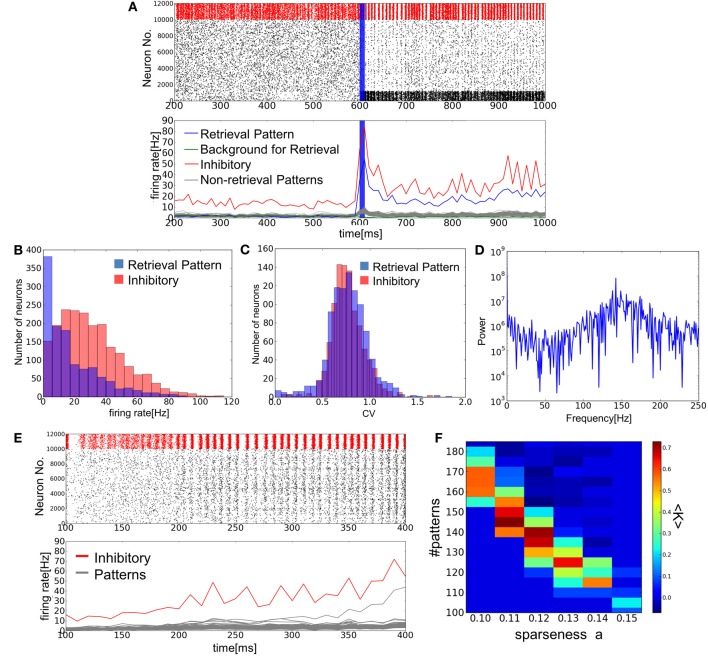
**Simulation of memory retrieval in the associative memory model.** Here, *a* = 0.12 and *p* = 140. **(A)** Raster plot and mean firing rates are shown. In the raster plot, red dots represent spikes of inhibitory neurons and black ones are spikes of excitatory neurons. PR neurons were displayed at the bottom. Blue vertical bars shows a period in which PR neurons were stimulated selectively by external inputs. The firing rates were calculated with a time bin of 10 ms. Gray lines are the firing rates of neurons encoding (*p*–1) non-retrieved memory patterns. **(B,C)** The distributions of firing rates and CVs for PR neurons and inhibitory neurons. **(D)** The power spectrum of inhibitory neural activity. We used the firing rates that were calculated with time bins of 1 ms. The results shown in **(B), (C)**, and **(D)** were calculated from the simulated spike data in the interval 650 ms <*t*<1600 ms shown in **(A)**. The average firing rates of BG neurons are too low (0.9 Hz) to calculate the distributions of firing rates and CVs. **(E)** Simulation results are shown at *a* = 0.12 and *p* = 110. Note that the value of *p* is smaller than the one used in **(A)**, and one of memory patterns was activated without a cue signal around 300 ms. **(F)** The average success rate <*K*> was numerically obtained for various values of *p* and *a*.

Figure 3A shows an example of successful memory retrieval by the network model. Without external input, the model is able to sustain spontaneous activity in which both PR neurons and BG neurons fire at low firing rates. If PR neurons (encoding memory pattern 1) are innervated by a brief cue signal (Materials and Methods), the model changes its dynamic behavior from spontaneous activity to a retrieval state, in which the average firing rates of the corresponding PR neurons are much higher than those of BG neurons. In fact, the firing rates of BG neurons are lower than the average firing rate of all excitatory neurons. As explained later, the network model can retrieve a memory pattern successfully if the number of stored patterns is within a certain range between upper and lower critical values. When this number is close to the upper bound (i.e., the storage capacity), excitatory neurons show sparse irregular firing in the retrieval state (Figures 3B,C). Inhibitory neurons also exhibit high coefficients of variations (CVs), although they fire near-synchronously at relatively high firing rates (Figure 3D).

When the number of stored patterns is smaller than a lower critical value, the model does not have a stable spontaneous activity. Due to the intrinsic noise generated by recurrent synaptic input, the network state eventually evolves from the resting state into one of the embedded patterns (Figure 3E). Therefore, the resting state is only quasi-stable. Furthermore, the memory patterns evoked in the final network state depend neither on the initial state nor cue signal. This is presumably because the state transition almost always targets such memory patterns that are separated by lower potential barriers from the resting state than other patterns. Therefore, the other memory patterns are difficult to recall and the model is unable to perform AM. If *p* is close to, but larger than the lower critical value, the network model remains in the initial resting state for more than hundreds of milliseconds, much longer than the membrane time constant. This long lifetime of the resting state indicates that the state transition is caused by stochastic fluctuations in network activity. Even if the spontaneous activity state no longer exists, the network could show slow dynamics due to the flatness of the potential function around the ghost of spontaneous activity state. In this case, the lifetime of the (ghost) spontaneous state would not be distributed exponentially. In practice, it is difficult to find the exact critical value by numerical simulations or by the present mean-field approximation, which does not explicitly take into account the influences of non-retrieved memory patterns.

Regarding the trial average <*K*> of the parameter *K*, which was evaluated over 15 trials on each value of parameters, as the success rate of retrieval, we display the areas of the two-dimensional parameter space spanned by *p* and *a* in which <*K*> is greater than 50% (Figure 3F). Given the value of *a*, the network model can retrieve a memory pattern successfully only if the number of stored patterns is between upper and lower critical values, where the upper bound represents the critical storage capacity (Hopfield, 1982; Amit et al., 1985). Interestingly, the retrieval states become unstable also in the regime of low memory load. This property is characteristic to the models that replicate spontaneous activity (Curti et al., 2004; Latham and Nirenberg, 2004; Roudi and Latham, 2007), and is not seen in other AM networks that do not have such a global state. An intriguing property of our model is that it generates spontaneous activity internally by reverberating synaptic input without external noise. We will demonstrate the non-trivial effect of internal noise later in detail.

### Role of the subthreshold membrane potentials in memory recall

Long-tailed distributions of EPSPs were previously shown to achieve stochastic resonance between postsynaptic firing and presynaptic spikes at sparse strong recurrent synapses in asynchronous irregular states of cortical networks (Teramae et al., 2012). In this stochastic resonance, the average membrane potentials of individual neurons are maintained at a subthreshold level optimal for spike transmission by very strong synapses. Below, we demonstrate how the present network controls the subthreshold membrane potential dynamics of individual neurons for efficient memory recall.

**Figure 4:**
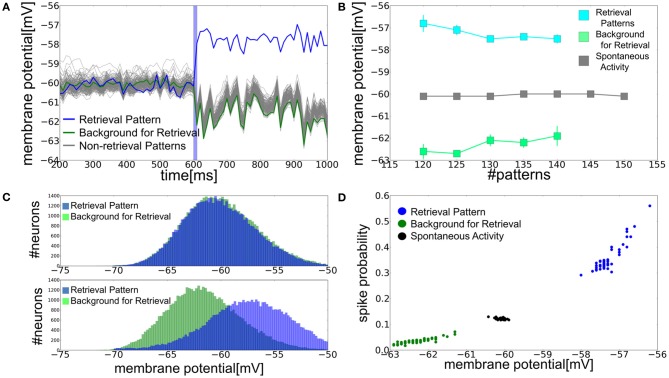
**Role of the subthreshold membrane potential distributions in memory retrieval. (A)** Time evolution of the average membrane potential is shown for *a* = 0.12 and *p* = 140. Blue vertical bar shows the duration of stimulus presentation as in Figure 3A. **(B)** Relationship between the average membrane potential and the number of stored patterns at *a* = 0.12. Gray squares show the average membrane potential of all excitatory neurons in spontaneous activity state, whereas blue and green squares are the averages of PR neurons and BG neurons in retrieval states. Error bars show the standard deviations of the trial variability. **(C)** Distributions of the membrane potential were calculated for spontaneous and retrieval states. In calculating the distributions for BG neurons, *N*_*E*_ a neurons were randomly chosen for the normalization. Peaks at *v* = −70 mV, which arose from the refractory period, were removed from the distributions. **(D)** Relationships between the spiking probability and average membrane potential. We calculated the spiking probability of a postsynaptic neuron from 1 to 5 ms after the firing of the presynaptic neurons sending strong inputs (EPSP >6 mV) to the post-synaptic neuron.

In the parameter regime of the bistable network dynamics, the average membrane potentials of PR and BG neurons split into two distinct levels in the retrieval state: the average membrane potentials are more depolarized in PR neurons than in BG neurons (Figure 4A). The differences in the average membrane potentials between the two categories of neurons become smaller as the memory load becomes heavier (Figure 4B). In the spontaneous firing state, distinctions between PR neurons and BG neurons are merely formal and they have identical Gaussian-like membrane potential distributions with the same means and variances. In the retrieval state, the membrane potentials of PR and BG neurons also obey Gaussian-like distributions with approximately the same variances. However, their means are clearly different for the two classes of neurons (Figure 4C). This splitting of the EPSP-amplitude distributions has significant implications in memory retrieval. The amplitude of EPSPs is typically larger than 6 mV at very strong synapses. This means that 10% of neurons can fire in the spontaneous firing state when a presynaptic spike arrives at a very strong synapse. By contrast, in the retrieval state the membrane potential distributions are shifted toward a more depolarized level in PR neurons (*v*_*i*_ > −56 mV), hence their probability of firing in response to such a presynaptic input increases by more than 30%. On the contrary, BG neurons rarely have membrane potentials higher than −56 mV, so they show only a very small firing probability for input at a strong synapse (Figure 4D).

### Comparison with the mean-field approximation

To clarify the dynamical properties of the present model, we conducted an analytical study using the mean-field approximation (Materials and Methods) and examined the validity of the analytical results by numerical simulations. In the mean-field approximation, we deal with the retrieval of a representative memory pattern by regarding other patterns as a noise reservoir. In this approximation, the model consisted of the three neuronal populations, i.e., excitatory PR neurons, excitatory BG neurons, and inhibitory neurons (Figure 2A). Below, the indices *p* and *b* refer to any quantity related to PR or BG neurons, respectively.

To investigate the dynamical phases of the model, we derived the nullclines of the average firing rates of PR and BG neurons by setting as ṙ_*p*_ = ṙ_*b*_ = 0 in Equations (12)–(15). In doing so, we assumed that inhibitory neurons are enslaved by excitatory neurons. In deriving the nullclines, we used parameters *r*_*Io*_ and *k*_*o*_ to fit the balanced firing rates obtained in numerical simulations (Figure 2B) and derived the averages and variances 〈*G*_*qq*′_〉 and 〈*G*^2^_*qq*′_〉 (*q, q*′ = *p, b, E*) from numerically generated Hebbian connection matrices *G*_*ij*_. We evaluated the contributions of the long tail of synaptic connections to the mean-field equations by taking into account the contributions of *N*_*s*_ strong-sparse connections and those of weak-dense synapses separately. This number remains to be a free parameter that should be fixed at a reasonable value. We found that the choice of *N*_*s*_ = 3 and the EPSP amplitude of *V*_*s*_ = 6.5 mV for these synapses yield an excellent agreement between the analytical and numerical results.

**Figure 5:**
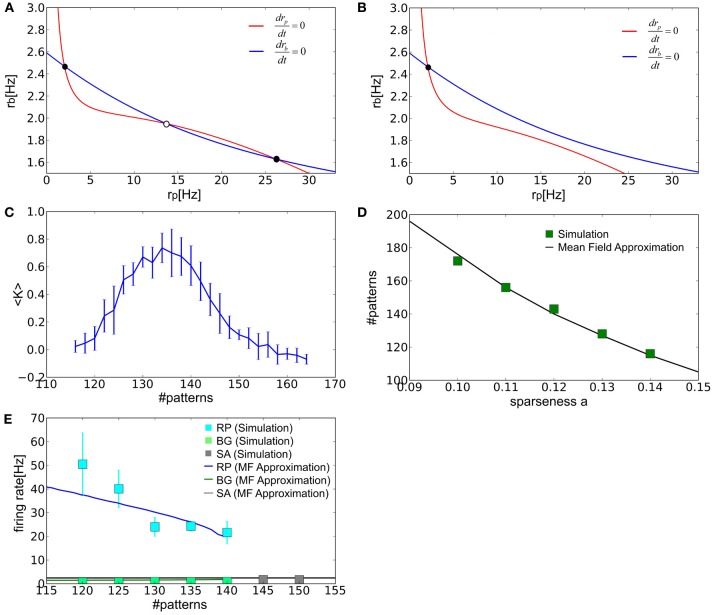
**The mean-field analysis of the equilibrium states. (A,B)** The nullclines of *r*_*p*_ (red) and *r*_*b*_ (blue) are shown for the values of *p* that are well below or greater than the critical storage capacity. Filled and empty circles show stable and unstable fixed points, respectively. **(C)** Relationship between <*K*> and the number of stored patterns is plotted. Error bars represent SD. We calculated the average value of of *K*_μ_ over different patterns μ in a given network and then averaged the value over different realizations of the network (i.e., the connection matrix). Therefore, 〈*K*〉 = ∑^*k*_*max*_^_*k*_ ∑^μ_*max*_^_μ_ K^(*k*)^_μ_/(*k*_*max*_μ_*max*_) and the standard deviation $\sigma =\sqrt{1/k_{max}{\sum_{k}^{k_{max}}\left(1/\mu_{max}\sum_{\mu }^{\mu_{max}}K_{\mu }^{\left(k\right)}-\langle K\rangle \right)}^{2}}$, where index *k* runs over the different realizations and *k*_*max*_ = μ_*max*_ = 10. **(D)** The storage capacity was evaluated numerically and analytically as a function of the sparseness parameter. **(E)** The average firing rates of all excitatory neurons were calculated in various states at *a* = 0.12. “SA” labels spontaneous activity.

When the number of stored patterns is in an adequate range, the nullclines of *r*_*p*_ and *r*_*b*_ yield three intersection points corresponding to the fixed points of the network dynamics in the two-dimensional space spanned by the parameters. According to a linear stability analysis, the central fixed point is unstable and the two fixed points on both sides are stable. Therefore, a stable fixed point in the regime of low or high *r*_*p*_ corresponds to spontaneous activity and the retrieval state, respectively (Figure 5A). As the number of stored patterns is increased, the retrieval state disappears at a critical value through a saddle-node bifurcation (Figure 5B). As a result, the system has only one stable state corresponding to spontaneous activity.

We can also determine the storage capacity by numerical simulations. As more patterns are stored the value of <*K*> gradually decreases until it finally vanishes (Figure 5C). The value of <*K*> is close to 0.5 in the vicinity of the critical point. Therefore, we may define the storage capacity as the number of patterns for which <*K*> is 0.5. The values of the storage capacity thus evaluated numerically and analytically are shown in Figure 5D as a function of the sparseness parameter. In Figure 5E, we also calculated the average firing rates of spontaneous activity *r*_*E*_ and those of PR and BG neurons in the retrieval state for *a* = 0.12. In all cases, the numerical and analytical treatments show a reasonably good agreement.

### Network dynamics at low memory load

As mentioned previously, this model also has a lower bound for the memory load in addition to an upper bound or the storage capacity. This instability reflects the fact that the model generates internal noise for memory recall by weak-dense synapses. Below, we investigate this instability by means of the mean-field approximation.

Because fluctuations in *r*_*b*_ are much smaller than those in *r*_*p*_, we may replace *r*_*b*_ with its fixed point r^*^_b_ in the mean-field analysis. Then, the dynamics of the model is approximately described with a single parameter *r*_*p*_ as:

(16) $$\frac{dr_{p}}{dt}=-\frac{1}{\tau_{f}^{p}}\left(r_{p}-{\hat{r}}_{p}\left(r_{p},r_{b}^{*}\right)\right)=-\frac{dU\left(r_{p}\right)}{dr_{p}}$$

**Figure 6:**
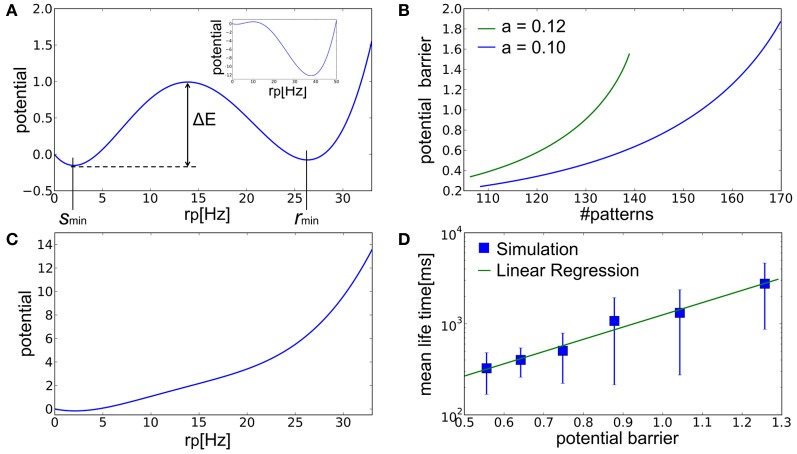
**The landscape of the potential fucntion *U*(*r*_*p*_). (A)** The potential function calculated at *a* = 0.12 and *p* = 135 has two local minima at *r*_*p*_ = *s*_*min*_ and *r*_*min*_ corresponding to the spontaneous activity and retrieval state in Figure 5A, respectively. Inset displays a similar potential function for a smaller number of memory patterns (*a* = 0.12 and *p* = 120). **(B)** Relationships between the number of stored patterns and potential barrier Δ*E* that separates the spontaneous acivity state and the retrieval state for different values of sparseness. Here, *p* = 135. **(C)** The potential function calculated at *a* = 0.12 and *p* = 150 has only a single minimum corresponding to the stable fixed point in Figure 5B. **(D)** Relationship between the analytically calculated potential barrier and the mean life time obtained in simulations. Here, *a* = 0.1. The mean life time is the time the network took for escaping from initial resting states. Error bars show SD.

We calculated this potential *U*(*r*_*p*_) numerically. When the number of embedded patterns is in the range that enables memory recall, the potential *U*(*r*_*p*_) has two local minima corresponding to spontaneous activity and the retrieval state at *r*_*p*_ = *s*_*min*_ and *r*_*min*_, respectively (Figure 6A). As the number of stored patterns is decreased, the latter local minimum becomes deeper (Figure 6A, inset) and simultaneously the potential barrier Δ*E* separating the two local minima becomes lower (Figure 6B). Note that we measure Δ*E* always from the bottom of the potential, *U*(*s*_*min*_), in the present argument. The result implies that spontaneous activity cannot remain stable when the number of stored patterns becomes smaller. In this case, fluctuations in spontaneous activity make it easier for the network state to jump over the potential barrier into the retrieval state. On the contrary, when the number of stored patterns exceeds a critical value, the local minimum corresponding to the retrieval state disappears and the network ceases to function as AM (Figure 6C). Figure 6D displays the relationship between Δ*E* calculated by the mean-field approximation and the mean lifetime of spontaneous activity obtained by numerical simulations. The mean lifetime depends exponentially on Δ*E*, suggesting that the transition to the retrieval state is due to stochastic fluctuations around the spontaneous firing state.

While this model accomplishes a relatively high storage capacity with spiking neurons, it does not show a stable and robust retrieval of an arbitrary memorized activity when the number of stored memory patterns is small. While long-tailed EPSP distributions are known in neocortical and hippocampal circuits (Song et al., 2005; Lefort et al., 2009; Ikegaya et al., 2013), the instability of the model seems to be unrealistic in biological neural networks. Therefore, we explored a way to modify the model for circumventing this difficulty. We point out that the unrealistic property arises from the theoretical hypothesis that the weights of all excitatory synapses are determined solely by stored memory patterns. Therefore, we created a partly randomized connection matrix and studied the retrieval dynamics of a neural network with such synaptic connections by numerical simulations.

**Figure 7:**
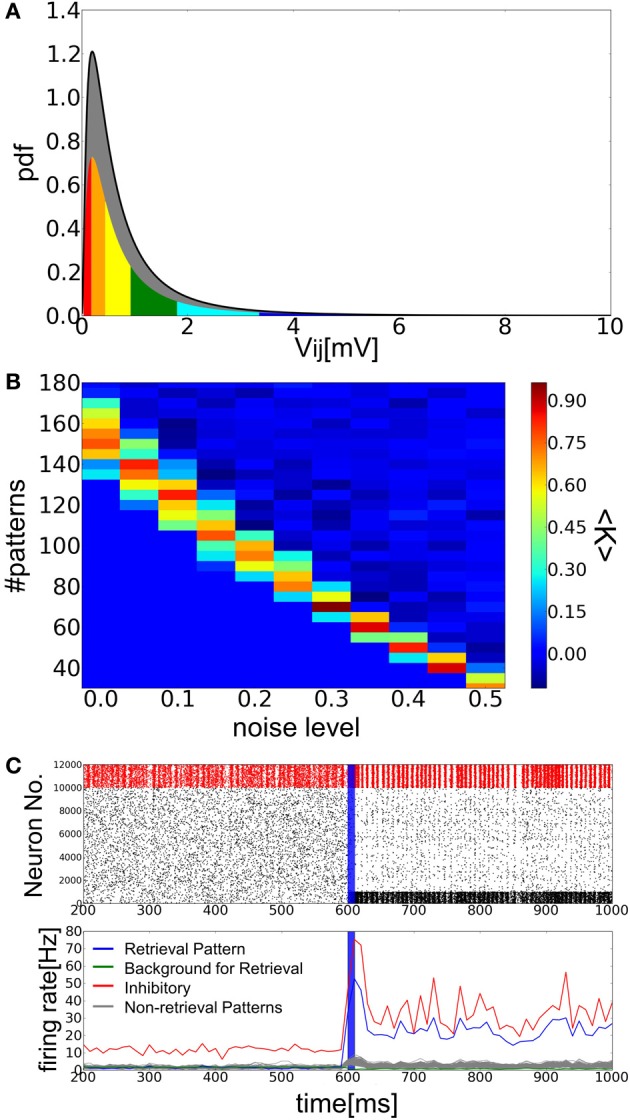
**Associative memory networks with Hebbian and random synaptic components. (A)** The distribution of the random components in synaptic weights for *c*_*r*_ = 0.4. The portion of synaptic connections used for storing memory patterns are colored as in Figure 1B, while the gray area shows the portion of synaptic connections not employed for the memory storage. **(B)** Phase diagram of the success rate <*K*> for various values of *c*_*r*_ and the number of embbed memory patterns *p*. **(C)** Raster plot and the time evolution of the averaged firing rates. Parameter values were *a* = 0.10, *c*_*r*_ = 0.2, and *p* = 100. Both panels were drawn in the same way as in Figure 3A.

We define partly randomized EPSP amplitudes *V*^*c*_*r*_^_*ij*_ of E-to-E synapses as follows:

(17) $$V_{ij}^{c_{r}}=\{\begin{array}{cc} v_{ij}\sim logN(\mu ,\sigma ) & if\;\eta_{ij}<c_{r}\\ V_{ij} & otherwise \end{array}$$

where *V*_*ij*_ is the EPSP amplitude constructed previously from the long-tailed EPSP distribution, *c*_*r*_ is the fraction of the randomized synapses in all E-to-E synapses (0 < *c*_*r*_ < 1), and η_*ij*_ is a random variable that takes a value between 0 and 1 (Figure 7A). Here, *c*_*r*_ cannot be too large to maintain reasonably large memory components in the connection matrix. The conductance constants were set as *G*^*EI*^ = 0.016, *G*^*IE*^ = 0.0018, and *G*^*II*^ = 0.002, and the other parameters took the same values as in the previous simulations. If we increase the noise fraction *c*_*r*_ for a small number of memory patterns, the retrieval state is also stabilized with a small memory load (Figure 7B). Spiking activity of the modified model during memory retrieval is similar to that of the previous model (Figure 7C). Thus, the inclusion of noisy components in synaptic connections secures the stability of retrieval dynamics with Hebbian synapses obeying long-tailed EPSP amplitude distributions.

## Discussion

We have presented an AM network model of spiking neurons and explored its dynamical properties during memory retrieval. According to results of recent electrophysiological studies (Song et al., 2005; Lefort et al., 2009; Ikegaya et al., 2013), we constructed Hebbian synaptic connetions that obey a long-tailed or a lognormal distribution of synaptic weights. Our model possesses a spontaneous firing state with low frequency neuronal firing (~1 Hz) and multiple persistent states with high frequency neuronal firing (~30 Hz), in which one of the embedded memory patterns is recalled.

### Implications of long-tailed weight distributions in stochastic network dynamics

Several papers have already studied the implications of long-tailed distributions of EPSP amplitudes in the dynamics of local cortical networks. Such EPSP-amplitude distributions account for a long-tailed distribution of firing rates in spontaneous firing of *in vivo* cortical neurons (Koulakov et al., 2009). The distribution contains many weak synapses and a small number of extremely strong synapses, and we recently showed that this coexistence of dense-weak and sparse-strong synapses enables recurrent cortical networks to generate an autonomous activity with highly irregular sparse firing of single neurons (Teramae et al., 2012). This persistent activity replicated various statistical properties of spontaneous cortical activity observed in experiment. We demonstrated that long-tailed weight distributions generate intrinsic noise optimal for spike communications by individual neurons. In short, the stochastic resonance effects generated by massively many weak synapses facilitate the faithful transmission of spike information received at strong synapses. Ikegaya et al. (2013) recorded long-tailed weight distributions also in the hippocampus and constructed a recurrent network model based on their experimental findings. This model involved NMDA receptors at recurrent synapses, which were crucial for stabilizing spontaneous activity in the model. The present model, however, does not involve NMDA receptors as the slow synaptic current is unnecessary for the genesis of spontaneous activity (Teramae et al., 2012). Finally, we note that long-tailed distributions of synaptic weights are well consistent with STDP (Gilson and Fukai, 2011).

### Comparison with previous spiking models of associative memory

Several models have been proposed to study the dynamics of memory retrieval in AM networks of integrate-and-fire type neurons (Sommer and Wennekers, 2001; Latham and Nirenberg, 2004; Curti et al., 2004; Aviel et al., 2005; Roudi and Latham, 2007). These models typically store sparsely coded memory patterns, for which AM networks in general exhibit a good performance. In comparison with AM networks of binary neurons, however, the networks of spiking neurons can generally store a relatively small number of memory patterns (Curti et al., 2004; Latham and Nirenberg, 2004; Roudi and Latham, 2007). Though, to our knowledge, no rigorous proof has been known, this low performance presumably reflects the leaky property of synaptic integration by spiking neurons. This is an interesting difference between the two classes of neural network models. It is also often difficult to maintain the firing rates of PR and BG firing neurons within a physiologically reasonable range, while keeping irregular spiking as typically observed in *in vivo* cortical neurons.

It is worthwhile to compare the storage capacity between different AM models of spiking neurons. A regorous comparision seems to be difficult since the retrieval performance of such models often shows a strongly non-linear dependence on parameter values. However, the storage capacity in general increases with the sparseness of memory patterns and decreases with the sparseness of synaptic connectivity. Therefore, the parameter α = (*pa*|ln *a*|/*c*_*E*_*N*_*E*_) may be used for measuring the storage capacity (or the information capacity) of AM models with similar sparseness and network size. The value of this parameter is 0.036 (*N*_*E*_ = 10,000, *c*_*E*_ = 0.1, *a* = 0.12, *p* = 140) in our model, 0.0058 (*N*_*E*_ = 8000, *c*_*E*_ = 0.25, *a* = 0.1, *p* = 50) in Latham and Nirenberg (2004), and 0.0056 (*N*_*E*_ = 8000, *c*_*E*_ = 0.2, *a* = 0.05, *p* = 60) in Curti et al. (2004). Therefore, out model has a larger storage capacity than the other models of spiking neurons in similar ranges of parameter values.

This model shows spontaneous firing of neurons, or BG activity, without any external input. As shown in our previous model of spontaneous cortical activity (Teramae et al., 2012), the coexistence of sparse-strong and dense-weak synaptic connections on each neuron enables individual neurons to faithfully transmit presynaptic spikes at sparse-strong synapses by means of stochastic resonance. In other words, the model utilized dense-weak recurrent synapses to generate internal noise optimal for this resonance effect. Input to dense-weak synapses maintains a depolarized subthreshold membrane potential and input to sparse-strong synapses evoke spikes with a certain probability. Thus, the broad range of synaptic strength enables the individual neurons to maintain spontaneous firing at low firing rates.

Our study has further shown that internal noise can selectively sustain the persistent firing of neuronal subgroups when synaptic connections are determined by the Hebbian rule. Due to relatively strong recurrent connections between PR neurons, they receive intense noise via weak-dense synapses when they are activated. This noise strongly depolarizes their membrane potentials and consequently these neurons fire with high probabilities in response to a spike input at sparse-strong synapses. By contrast, BG neurons have lower subthreshold membrane potentials and lower probabilities of spike responses to strong inputs. This probabilistic spiking achieved by the cooperation of SSWD synaptic inputs may provide a novel mechanism of probabilistic neural computations.

It was previously pointed out that spontaneous firing states turn to be unstable in AM network models of spiking neurons when the number of stored memory patterns is small (Curti et al., 2004). It is also known that when synaptic connections are described as the sum of Hebbian components and random components, the Hebbian components should be sufficiently small to maintain stable spontaneous firing states (Latham and Nirenberg, 2004; Roudi and Latham, 2007). The present model also exhibits the instability of spontaneous firing states when the memory load is too low. According to the previous results, we demonstrated that the addition of random components to lognormally distributed Hebbian components in the connection matrix removes the instability from the model.

The genesis of highly irregular asynchronous states is not trivially easy in modeling persistent activity of recurrent neural networks. Such activity is typically seen in working memory, and various solutions to this problem have been proposed, which include the uses of slow reverberating synaptic current (Wang, 1999), balanced excitatory and inhibitory synaptic input (Renart et al., 2007; Roudi and Latham, 2007; Mongillo et al., 2012), short-term synaptic depression (Barbieri and Brunel, 2007; Mongillo et al., 2012), and modular network structure (Lundqvist et al., 2010). In this study, we have shown that long-tailed distributions of EPSP amplitudes generate a highly irregular persistent activity in the memory retrieval, as was the case in spontaneous activity (Teramae et al., 2012).

In summary, we have developed an AM model of spiking neurons by taking long-tailed distributions of synaptic weights into account. Our network model exhibited excellent performance in the memory retrieval, suggesting an active role of internal noise in memory processing by the brain.

### Conflict of interest statement

The authors declare that the research was conducted in the absence of any commercial or financial relationships that could be construed as a potential conflict of interest.

## Appendix

### Derivation of fluctuations in synaptic inputs *s*^*Ep*^, *s*^*Eb*^

We define <*X*> as the averaging over both neural population and time bins. Amplitude fluctuations in excitatory synaptic inputs *s*^*Ep*^ on PR neurons are represented as:

$$\begin{array}{l} {\left(s^{Ep}\right)}^{2}=\langle {\left(\sum_{j}^{N_{E}}c_{ij}G_{ij}^{pp}\sum_{s_{j}}\delta (t-s_{j}-d_{ij})-c_{E}N_{E}\left(a<G_{pp}>r_{p}+(1-a)<G_{bp}>r_{b}\right)\right)}^{2}\rangle \\ \;≅\langle {\left(\sum_{j}^{p}\left[c_{ij}G_{ij}^{pp}\sum_{s_{j}}\delta (t-s_{j}-d_{ij})-a<G_{pp}>r_{p}\right]\right)}^{2}\rangle +\langle {\left(\sum_{j}^{b}\left[c_{ij}G_{ij}^{bp}\sum_{s_{j}}\delta (t-s_{j}-d_{ij})-a<G_{bp}>r_{b}\right]\right)}^{2}\rangle . \end{array}$$

Thus, they can be separated into two components corresponding to input from PR neurons and that from BG neurons. For the former input,

(A1) $$\begin{array}{l} \langle {\left(\sum_{j}^{p}\left[c_{ij}G_{ij}^{pp}\sum_{s_{j}}\delta (t-s_{j}-d_{ij})-a<G_{pp}>r_{p}\right]\right)}^{2}\rangle ≅\langle \sum_{j}^{p}\left[{\left(c_{ij}-c_{E}\right)}^{2}{\left(G_{ij}^{pp}\right)}^{2}{\left(\sum_{s_{j}}\delta (t-s_{j}-d_{ij})\right)}^{2}\right]\rangle \\ \;+\langle \sum_{j}^{p}\left[c_{E}^{2}{\left(G_{ij}^{pp}-<G_{pp}>\right)}^{2}{\left(\sum_{s_{j}}\delta (t-s_{j}-d_{ij})\right)}^{2}\right]\rangle +\langle \sum_{j}^{p}\left[c_{E}^{2}<G_{pp}{>}^{2}{\left(\sum_{s_{j}}\delta (t-s_{j}-d_{ij})-r_{p}\right)}^{2}\right]\rangle \end{array}$$

(A2) $$≅N_{E}a[c_{E}(1-c_{E})<G_{pp}^{2}>r_{p}+\;c_{E}^{2}\left(<G_{pp}^{2}>-<G_{pp}{>}^{2}\right)r_{p}+c_{E}^{2}<G_{pp}{>}^{2}r_{p}]$$

(A3) $$≅c_{E}N_{E}a<G_{pp}^{2}>r_{p}.$$

In the calculation of Equation (A3), we assume that the input is in the regime of low firing rates. Therefore, 〈∑_*s*_*j*__ δ(*t* − *s*_*j*_ − *d*_*ij*_)〉 ≃ 0 or 1, and

(A4) $$\langle {\left(\sum_{s_{j}}\delta (t-s_{j}-d_{ij})\right)}^{2}\rangle ≅\langle \sum_{s_{j}}\delta (t-s_{j}-d_{ij})\rangle .$$

Furthermore, we may assume that synaptic input is represented by Poisson spike trains. Under this assumption, the fluctuation of firing rates are written as,

(A5) $$\langle {\left(\sum_{s_{j}}\delta (t-s_{j}-d_{ij})-r_{p}\right)}^{2}\rangle ≅r_{p}$$

Input from BG neurons can be similarly calculated and the variance is evaluated as:

(A6) $${\left(s^{Eq}\right)}^{2}≅c_{E}N_{E}\left(a\langle G_{pp}^{2}\rangle r_{p}+\left(1-a\right)\langle G_{bp}^{2}\rangle r_{b}\right),$$

and (*s*^*Eb*^)^2^ is evaluated in a similar manner.

### Derivation of the population rate dynamics

In Gaussian approximation of synaptic inputs, the membrane potential *v*^*q*^_*i*_ is written as follows:

$$\begin{array}{l} \frac{dv_{i}^{q}}{dt}≅-\frac{1}{\tau_{e}^{q}}\left(v_{i}^{q}-V_{o}^{q}\right)-\eta_{i}^{Eq}\left(V_{o}^{q}-V_{E}\right)-\eta_{i}^{IE}\left(V_{o}^{q}-V_{I}\right),\\ \;\tau_{e}^{q}={\left[1/\tau_{m}^{E}+\langle g^{IE}\rangle +\langle g^{Eq}\rangle \right]}^{-1},\\ \;V_{o}^{q}=\tau_{e}^{q}\left[V_{L}/\tau_{m}^{E}+\langle g^{Eq}\rangle V_{E}+\langle g^{IE}\rangle V_{I}\right]. \end{array}$$

In the calculations below, we drop index *i* from equations as they are essentially the same for all excitatory neurons.

Let *v*^*q*^_*i*_ = *y*_*q*_, − η^*Eq*^_*i*_ (*V*^*q*^_*o*_ − *V*_*E*_) = *x*^*E*^_*q*_, −η^*Iq*^_*i*_ (*V*^*q*^_*o*_ − *V*_*I*_) = *x*^*I*^_*q*_, then the above equations are rewritten as:

$$\frac{dy_{q}}{dt}=-\frac{y_{q}}{\tau_{e}^{q}}+x_{q}^{E}+x_{q}^{I},\frac{dx_{q}^{E}}{dt}=-\frac{x_{q}^{E}}{\tau_{s}}+\sigma_{q}^{E}\zeta_{q}^{E}(t),\frac{dx_{q}^{I}}{dt}=-\frac{x_{q}^{I}}{\tau_{s}}+\sigma_{q}^{I}\zeta_{q}^{I}(t)$$

where (σ^*E*^_*q*_)^2^ = (*s*^*Eq*^)^*2*^ (*V*^*q*^_*o*_ − *V*_*E*_)^2^, (σ^*I*^_*q*_)^2^ = (*s*^*Iq*^)^2^ (*V*^*q*^_*o*_ − *V*_*I*_)^2^. Defining $z_{q}=-\frac{y_{q}}{\tau_{e}^{q}}+x_{q}^{E}+x_{q}^{I}$, we obtain

$$\frac{dy_{q}}{dt}=z_{q},$$

$$\frac{dz_{q}}{dt}=-\left(\frac{1}{\tau_{e}^{q}}+\frac{1}{\tau_{s}}\right)z-\frac{y}{\tau_{e}^{q}\tau_{s}}+\sqrt{{\left(\sigma_{q}^{E}\right)}^{2}+{\left(\sigma_{q}^{I}\right)}^{2}}\zeta_{q}(t).$$

Using Kramer's equation, we can derive the stationary probabilistic distribution *W*(*y, z*) for the above two variables as:

$$W(y,z)=\frac{\sqrt{\tau_{e}^{q}\tau_{s}}}{2\pi \sigma_{q}^{2}}\exp\left(-\frac{\tau_{e}^{q}\tau_{s}}{2\sigma_{q}^{2}}z^{2}-\frac{y^{2}}{2\sigma_{q}^{2}}\right),\sigma_{q}^{2}={\left[\frac{1}{\tau_{e}^{q}}+\frac{1}{\tau_{s}}\right]}^{-1}\frac{\tau_{e}^{q}\tau_{s}}{2}\left[\sigma_{Eq}^{2}+\sigma_{Iq}^{2}\right].$$

Then, we can calculate the output firing-rate of each neuron from the probabilistic current as:

(A7) $$r_{q}^{1}=\int_{0}^{\infty }zW(V_{th}-V_{o},z)dz=\frac{1}{2\pi \sqrt{\tau_{e}\tau_{s}}}\exp\left[-\frac{{\left(V_{th}-V_{o}^{q}\right)}^{2}}{2\sigma_{q}^{2}}\right].$$

An additional cause of the rate increases in postsynaptic neurons is the pool of extremely strong EPSPs, which contribute to the firing rate of each neuron by:

(A8) $$r_{q}^{2}=\frac{N_{s}r_{E}}{2}\left(\text{erf}\left[\frac{1}{\sqrt{2}\sigma_{q}}\left(V_{th}-V_{o}^{q}\right)\right]-\text{erf}\left[\frac{1}{\sqrt{2}\sigma_{q}}\left(V_{th}-V_{o}^{q}-V_{s}\right)\right]\right),$$

where *N*_*s*_ is the number of extremely strong synapses and *V*_*s*_ is the average EPSP size of these strong inputs. Thus, the overall firing rate is given as *r*_*q*_ = r^1^_*q*_ + *r*^2^_*q*_.
